# Pretreatment synthetic magnetic resonance imaging predicts disease progression in nonmetastatic nasopharyngeal carcinoma after intensity modulation radiation therapy

**DOI:** 10.1186/s13244-023-01411-y

**Published:** 2023-04-05

**Authors:** Fan Yang, Haoran Wei, Xiaolu Li, Xiaoduo Yu, Yanfeng Zhao, Lin Li, Yujie Li, Lizhi Xie, Sicong Wang, Meng Lin

**Affiliations:** 1 Department of Diagnostic Radiology, National Cancer Center/National Clinical Research Center for Cancer/Cancer Hospital, Chinese Academy of Medical Sciences and Peking Union Medical College, Beijing, 100021, China; 2 MR Research China, GE Healthcare, Beijing, China

**Keywords:** Synthetic magnetic resonance imaging (SyMRI), Nasopharyngeal carcinoma (NPC), Treatment outcome

## Abstract

**Background:**

To investigate the potential of synthetic MRI (SyMRI) in the prognostic assessment of patients with nonmetastatic nasopharyngeal carcinoma (NPC), and the predictive value when combined with diffusion-weighted imaging (DWI) as well as clinical factors.

**Methods:**

Fifty-three NPC patients who underwent SyMRI were prospectively included. 10th Percentile, Mean, Kurtosis, and Skewness of T1, T2, and PD maps and ADC value were obtained from the primary tumor. Cox regression analysis was used for analyzing the association between SyMRI and DWI parameters and progression-free survival (PFS), and then age, sex, staging, and treatment as confounding factors were also included. C-index was obtained by bootstrap. Moreover, significant parameters were used to construct models in predicting 3-year disease progression. ROC curves and leave-one-out cross-validation were used to evaluate the performance and stability.

**Results:**

Disease progression occurred in 16 (30.2%) patients at a follow-up of 39.6 (3.5, 48.2) months. T1_Kurtosis, T1_Skewness, T2_10th, PD_Mean, and ADC were correlated with PFS, and T1_Kurtosis (HR: 1.093) and ADC (HR: 1.009) were independent predictors of PFS. The C-index of SyMRI and SyMRI + DWI + Clinic models was 0.687 and 0.779. Moreover, the SyMRI + DWI + Clinic model predicted 3-year disease progression better than DWI or Clinic model (*p* ≤ 0.008). Interestingly, there was no significant difference between the SyMRI model (AUC: 0.748) and SyMRI + DWI + Clinic model (AUC: 0.846, *p* = 0.092).

**Conclusion:**

SyMRI combined with histogram analysis could predict disease progression in NPC patients, and SyMRI + DWI + Clinic model further improved the predictive performance.

**Supplementary Information:**

The online version contains supplementary material available at 10.1186/s13244-023-01411-y.

## Key points

1. SyMRI showed excellent performance in predicting disease progression in NPC.
2. T1_Kurtosis and ADC were independent predictors of progression-free survival.
3. T1_Kurtosis was lower in the non-disease progression group than the disease progression group.
4. Combination of SyMRI, DWI, and clinical factors could improve the predictive performance.

**Supplementary Information:**

The online version contains supplementary material available at 10.1186/s13244-023-01411-y.

## Introduction

Nasopharyngeal carcinoma (NPC) is an aggressive head and neck cancer and more than 70% of new patients were diagnosed in East and Southeast Asia [1]. Intensity-modulated radiation therapy (IMRT) with or without chemotherapy is the mainstay of treatment for NPC. Despite good overall survival after treatment, approximately 10–30% of patients would occur locoregional relapse or distant metastases [2, 3]. Moreover, retreatment for disease progression is challenging due to a lack of effective means and fatal complications. Thus, it is critical to find high-risk patients for disease progression before treatment.

Pretreatment TNM stage is most commonly used and is the benchmark to establish treatment regimens, while large variations are reported in the treatment response and clinical outcomes of patients with the same stage under similar treatment [4]. Ignored intratumor characteristics and heterogeneity may be its major limitation [5]. Nowadays, circulating EBV DNA [6–8], serum lactate dehydrogenase (LDH) [9], C-reactive protein (CRP) [10], and systemic immune-inflammation index (SII) [11] have been demonstrated to influence recurrence and survival in NPC patients. Notably, the addition of pretreatment EBV DNA into the 8th Edition TNM stage system greatly improved its prognostic performance [6–8]. However, even using the same assay and identical procedures, comparatively large interlaboratory variability was found in research works [12]. Therefore, constructing a robust prognostic predictor is of vital importance.

Magnetic resonance imaging (MRI) is the commonly used imaging modality for NPC diagnosis, clinical staging, and therapy monitoring. However, conventional MRI such as T1-weighted imaging (T1WI) and T2-weighted imaging (T2WI) only reflect morphological characteristics, resulting in insufficient diagnostic performance in therapy assessment and survival prediction [13]. Recently, advanced MRI techniques including intravoxel incoherent motion diffusion-weighted imaging (IVIM-DWI), dynamic contrast-enhanced, arterial spin labeling, as well as amide proton transfer (APT) have been utilized to evaluate the early efficacy of chemoradiotherapy and higher apparent diffusion coefficient (ADC), pure diffusion coefficient and APT values were correlated with disease progression in NPC [14–16].

Synthetic MRI (SyMRI), using multi-delay and multi-echo (MDME) sequence, could generate quantitative T1, T2, and proton density (PD) maps and multi-contrast qualitative images (including T1WI, T2WI, and PDWI) within the clinically feasible time. The research [17] in head and neck region proved the clinical feasibility of generating synthetic T1WI and T2WI images from quantitative relaxometry mapping. Recently, preliminary SyMRI studies have demonstrated a potential role in evaluating prognostic factors in breast [18, 19], prostate [20], bladder [21], and rectal cancer [22, 23]. However, only short-term outcome on tumor was researched and the value of SyMRI on NPC prognostic assessment has not been investigated. Therefore, the purpose of our study is to investigate the predictive value of SyMRI in the long-term prognostic assessment of nonmetastatic NPC patients. In addition, the diagnostic performance of SyMRI was also compared with combined SyMRI, DWI, and clinical factors.

## Methods

### Patients

**Fig. 1:**
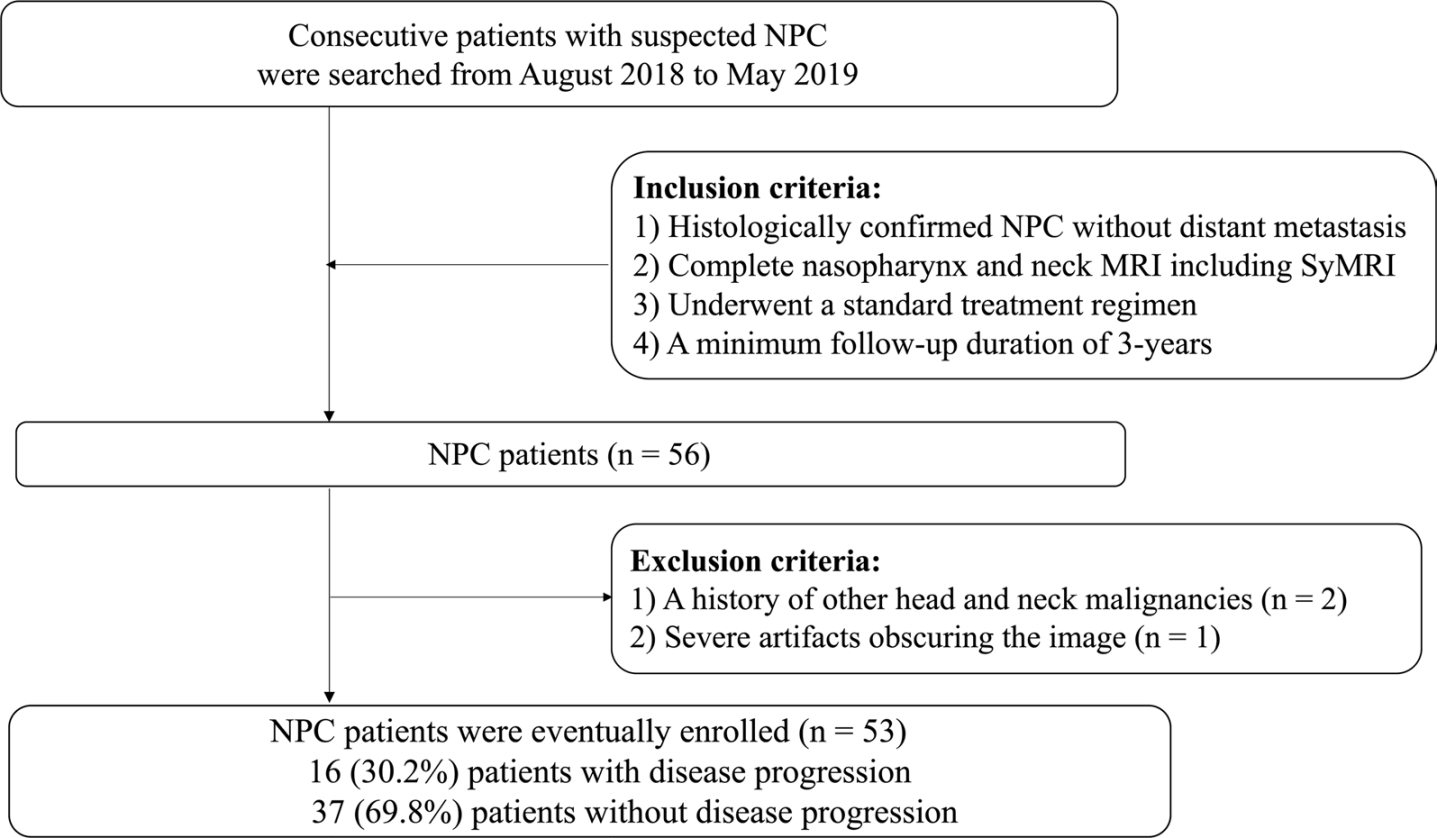
Workflow diagram of patient selection

This prospective study was approved by the Ethics Committee of our hospital, and informed consent was obtained from all patients before MRI examination. In total, 62 consecutive patients with primary NPC between August 2018 and May 2019 were evaluated for inclusion in this study. The inclusion criteria was as follows: (1) Histologically confirmed NPC without distant metastases. (2) Complete nasopharynx and neck MRI including SyMRI before treatment. (3) Underwent a standard treatment regimen that consisted of IMRT and/or chemotherapy based on TNM classification. (4) Surviving patients with a minimum follow-up duration of 3 years. The exclusion criteria included (1) A history of other head and neck malignancies; (2) Severe motion artifacts on MRI. Ultimately, 53 patients were recruited and the workflow diagram of the study cohort is shown in Fig. 1.

Patients underwent conventional imaging for staging workup including nasopharynx–neck MRI, neck–chest–abdomen–pelvis CT, neck ultrasonography, and/or fluorodeoxyglucose positron emission tomography-computed tomography (FDG-PET/CT). Two senior radiologists (M.L. and X.Y. with 21 and 18 years of diagnostic radiology experience, respectively) performed TNM staging based on medical records and images according to the American Joint Committee on Cancer (AJCC) Cancer staging Manual Eighth Edition, with any disagreement resolved through discussion.

### Imaging acquisition

All MRI examinations were performed on a 3 T scanner (Pioneer, GE Healthcare, USA), with an 8-channel head and neck phased array coil. Conventional MRI sequences including axial T1WI, T2WI with fat suppress, DWI, and enhanced axial T1WI with fat suppress were acquired according to the following parameters: repetition time, 482, 6100, 2930, 250; echo time,13.6, 85, 80, 13.6; the field of vision, 26; acquisition matrix, 256 × 320, 256 × 288, 128 × 96, 256 × 320; slice thickness/gap, 4.0/0.4; number of excitations, 2, 2, 4, 2; acquisition time (min), 4.02, 4.41, 1.22, 3.47. For SyMRI, the parameters were as follows: repetition time, 6200; echo time, 18.9/94.7; the field of vision, 26; acquisition matrix, 256 × 320; slice thickness/gap, 4.0/0.4; number of excitations, 1; acquisition time, 7.02.

### Data processing

The acquired raw images were processed using SyMRI software (version 8.0, Synthetic MR, Linkoping, Sweden) to generate three quantitative maps (T1 map, T2 map, and PD map) and multiple contrast-weighted images (T1WI, T2WI, T1WI FLAIR, T2WI FLAIR, PDWI and so on). Then, two radiologists (X.Y., F.Y., 18 and 3 years of tumor-imaging experience) manually delineated volumes of interest (VOIs) of the primary tumor on SyT2WI images using ITK-SNAP software (version 2.2.0, www.itksnap.org), with reference to conventional images and excluding any visible necrosis and hemorrhage. The VOIs were automatically copied to the T1 map, T2 map, and PD map to extract histogram features, including Mean, 10th Percentile, Skewness, and Kurtosis using open-source PyRadiomics (http://www.radiomics.io/pyradiomics.html). Additionally, referring to conventional images, the VOIs were delineated on the ADC maps, and mean ADC values were then obtained. Additionally, ten clinical factors, including patient age, sex, T stage, N stage, clinical stage, treatment, EBV DNA, LDH, CPR, and SII, were also analyzed.

### Treatment regimen

All included patients underwent the standard treatment regimen. Stage I was treated with IMRT alone, while stage II was treated with IMRT with or without chemotherapy. Stage III or IV was treated with IMRT and concurrent or adjuvant chemotherapy. The radiation doses of the primary tumor and enlarged lymph nodes are 66–74 Gy. The regions at risk of metastasis and bilateral cervical lymphatics were selectively irradiated to 50–60 Gy.

### Follow-up and outcome endpoints

After treatment completion, regular clinical and endoscopic examinations were performed every 3–4 months for the first 3 years, every 6 months during 4–5 years, then yearly. Patients with suspected disease relapse underwent imaging examination (including CT, MRI, ultrasonography, bone scan, and PET/CT) and/or biopsy through histopathological examination. The clinical outcome of this study was progression-free survival (PFS), which was defined as the time from the start of treatment to the date of disease progression (local–regional recurrences or distant metastases), death, or last follow-up, whichever occurred first.

### Statistical analysis

All statistical analyses were performed using SPSS software (version 26.0, Chicago, IL), R (version 1.3.1073, R Foundation, Vienna, Austria), and X-tile (version 3.6.1, http://tissuearray.org). Interobserver consistency was analyzed using a two-way random interclass correlation (ICC), and histogram features with an ICC < 0.8 were excluded. A two-tailed *p* < 0.05 indicated statistical significance. Continuous data were expressed as mean and standard deviation and categorical variables were expressed as percentage. SyMRI parameters were compared between patients with and without disease progression using the independent sample-*t* test or Mann–Whitney U test, as appropriate. In consideration of the sample size of staging classification subgroups, the best groupings of staging classification based on the log-rank test (lowest *p* value) were used [24]. Univariate Cox proportional hazards regression analysis was performed to assess the correlation of SyMRI and DWI parameters with survival endpoints. Significant parameters were entered in a multivariable Cox proportional hazards regression analysis using the forward selection approach to construct models, and the relevant parameters were then reevaluated using confounding factors (age, sex, T stage, N stage, clinical stage, and treatment) [14, 16]. The optimal threshold values of significant parameters were obtained using X-tile software. The survival rate was calculated by using Kaplan–Meier analysis and differences were compared using log-rank test. The C-index and 95% confidence interval (95% CI) were obtained by bootstrap (*n* = 1000).

In addition, SyMRI and DWI parameters and clinical features that were associated with PFS were selected for classification models to identify whether disease progression has occurred at 3 years. The ROC curve was used to evaluate the predictive performance, and the area under the curve (AUC) comparisons were performed to determine the best predictive model by using the Delong test. The bootstrap test was applied. Moreover, leave-one-out cross-validation (LOOCV) was used to avoid over-fitting according to previous studies [25, 26]. In each round of the LOOCV, all study subjects except for one (testing set) were used as the training set, and the prediction error was assessed for the excluded test set. This procedure was repeated until each participant was tested once.

## Results

### Patient characteristics and study endpoints

The characteristics of the 53 NPC patients are given in Table 1. Ten of 53 (18.9%) patients were diagnostic with locoregional relapse, including primary tumor site in 7/53 (13.2%) patients and regional lymph nodes in 3/53 (5.7%) patients. Distant metastases (such as liver, bone, lung, and brain metastasis) were diagnosed in 8/53 (15.1%) patients; among them, 2/53 (3.8%) patients simultaneously occurred locoregional recurrence and distant metastases. The median follow-up duration for all patients was 39.6 (3.5, 48.2) months; for NPC patients with disease progression (*n* = 16) of 17.8 (3.5, 34.1) months; and for NPC patients without disease progression (*n* = 37) of 41.5 (38.6, 48.2) months. The 3-year PFS was 69.8%.

**Table 1:** Characteristics of NPC patients

Clinical characteristics	Disease progression group (*n* = 16)	Non-disease progression group (*n* = 37)	*p* value
Age	54.13 ± 8.45	48.08 ± 12.62	0.086
Gender			1.000
Male	13	29	
Female	3	8	
Histology			0.310
WHO II	8	13	
WHO III	8	24	
T stage			0.035
T1	0	10	
T2	6	5	
T3	5	14	
T4	5	8	
N stage			0.758
N0	1	6	
N1	6	11	
N2	4	9	
N3	5	11	
Clinical stage			0.847
I	0	1	
II	3	8	
III	5	11	
IV	8	17	
Treatment			1.000
IMRT	2	4	
IMRT + chemotherapy	14	33	
EBV DNA			0.311
< 4000	13	35	
≥ 4000	3	2	
LDH			0.686
< 182	9	23	
≥ 182	7	14	
SII			0.422
< 402.10	2	10	
≥ 402.10	14	27	
CRP			0.599
< 2.46	16	34	
≥ 2.46	0	3	

*IMRT* Intensity-modulated radiation therapy; *EBV* Epstein–Barr virus; *LDH* Lactate dehydrogenase; *SII* Systemic immune-inflammation index; *CRP* C-reactive protein

All parameters showed excellent inter-rater consistency (all ICC > 0.891, Additional file 1: Table S1). The details of 16 patients are attached in Additional file 1: Table S2.

### Correlation of imaging and clinical parameters with PFS

**Fig. 2:**
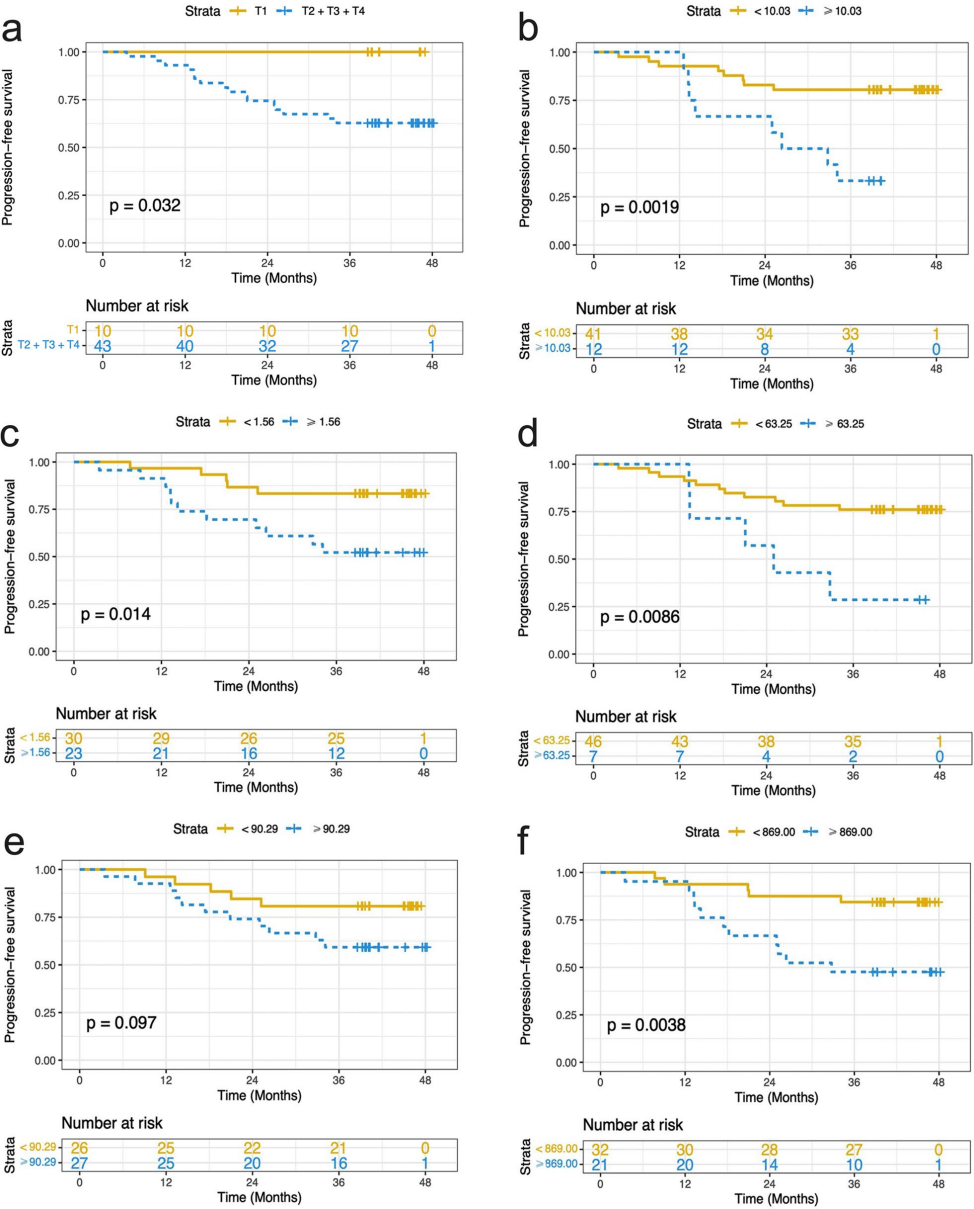
Kaplan–Meier plots of features that are associated with PFS and every plot has a log-rank *p* value and risk table. **a** T stage; **b** Kurtosis of T1 map; **c** Skewness of T1 map; **d** 10th Percentile of T2 map; **e** Mean of PD map; **f** Mean of ADC map. *PFS* Progression-free survival; *PD* Proton density; *ADC* Apparent diffusion coefficient

**Fig. 3:**
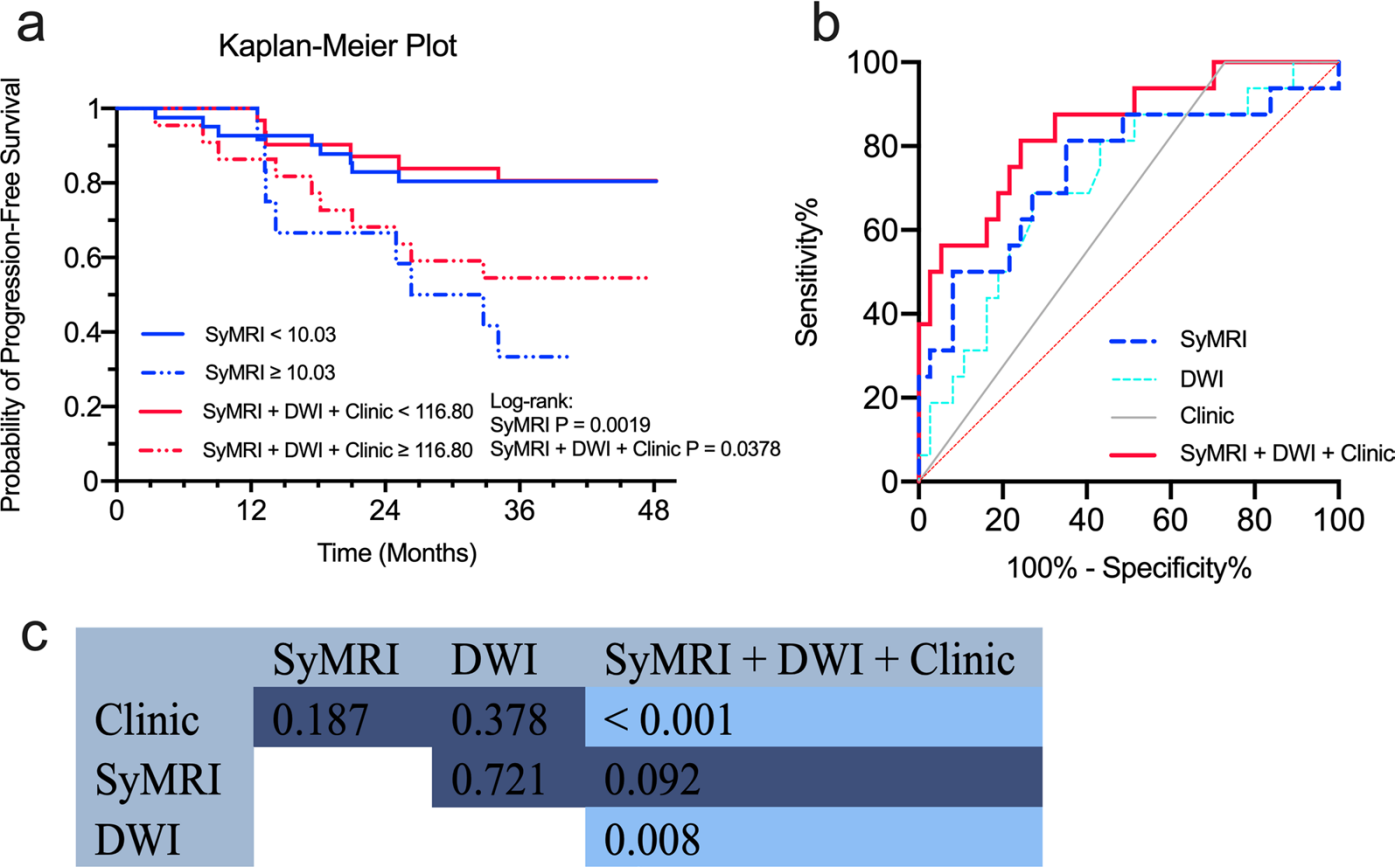
Performance of models and comparison between models. **a** Kaplan–Meier plots of the SyMRI model and SyMRI + DWI + Clinic model; **b** ROC curves of models in predicting 3-year disease progression; **c** The difference between different models using the DeLong test. *SyMRI* Synthetic magnetic resonance imaging; *DWI* Diffusion-weighted imaging; *ROC* Receiver operating characteristic

For staging classification, the best groupings were as follows: T1 and T2 + T3 + T4 (*p* = 0.032, Fig. 2), N0 and N1 + N2 + N3 (*p* = 0.425), and I + II and III + IV (*p* = 0.678). Among all clinical factors, only T stage was selected for the subsequent Clinic model. After univariate Cox regression analysis, high T1_Kurtosis and T1_Skewness, and low T2_10th and PD_Mean were correlated with poor PFS (*p* = 0.003, 0.024, 0.047, and 0.022, respectively) in NPC (Fig. 2, Table 2). In multivariate analysis, T1_Kurtosis remained a significant predictor of PFS, with HR of 1.083 (95% CI 1.007–1.165), whereas the associations with T1_Skewness, T2_10th, and PD_Mean were no longer significant (*p* > 0.05). After adjusting for confounding factors, results showed that T1_Kurtosis was an independent predictor of PFS (*p* = 0.043, Table 3). Additionally, multivariate Cox analysis also showed that ADC was an independent predictor of PFS (*p* = 0.002, Table 3). The Kaplan–Meier plot of the SyMRI model and SyMRI + DWI + Clinic model is shown in Fig. 3, and the C-index was 0.687 (95% CI 0.612, 0.762) and 0.779 (95% CI 0.716, 0.842), respectively. The bootstrap curves are presented in Additional file 1: Fig. S1.

**Table 2:** Cox analysis of SyMRI and DWI parameters for the prediction of disease progression in NPC patients

Parameters	HR (95% CI)	*p* value
*SyMRI*		
T1_10th	1.001 (0.996, 1.005)	0.825
T1_Mean	0.999 (0.996, 1.002)	0.571
T1_Kurtosis*	**1.083 (1.007, 1.165)**	**0.003**
**T1_Skewness**	**1.943 (1.090, 3.465)**	**0.024**
**T2_10th**	**0.929 (0.865, 0.999)**	**0.047**
T2_Mean	0.949 (0.883, 1.019)	0.146
T2_Kurtosis	1.000 (1.000, 1.001)	0.294
T2_Skewness	1.023 (0.978, 1.070)	0.326
PD_10th	0.889 (0.782, 1.011)	0.073
**PD_Mean**	**0.836 (0.717, 0.975)**	**0.022**
PD_Kurtosis	1.127 (0.821, 1.547)	0.460
PD_Skewness	1.111 (0.367, 3.367)	0.852
*DWI*		
**ADC**	**1.006 (1.001, 1.010)**	**0.002**

Parameters in bold mean* p* < 0.05; *Only T1_Kurtosis was selected to construct SyMRI model after multivariate Cox proportional hazards regression analysis

**Table 3:** Multivariable analysis for the prediction of PFS in NPC

Parameters	PFS (T1_Kurtosis and clinical factors)		PFS (ADC and clinical factors)	
	HR (95% CI)	*p* value	HR (95% CI)	*p* value
**T1_Kurtosis**	**1.093 (1.003, 1.192)**	**0.043**	**–**	**–**
**ADC**	–	–	**1.008 (1.002, 1.014)**	**0.007**
Age	1.040 (0.979, 1.192)	0.205	1.053 (0.994, 1.116)	0.078
Sex	1.446 (0.352, 5.937)	0.609	0.905 (0.238, 3.432)	0.883
Treatment	1.021 (0.085, 12.204)	0.987	0.429 (0.031, 5.984)	0.529
T stage	*	0.987	*	0.960
N stage	4.791 (0.484, 47.431)	0.180	1.963 (0.177, 21.820)	0.583
Clinical stage	0.262 (0.032, 2.123)	0.210	1.767 (0.203, 15.349)	0.606

Parameters in bold mean* p* < 0.05; * indicates the HR and 95% CI were not obtained because there was no disease progression in patients with T1 stage

*PFS* Progression-free survival; *NPC* Nasopharyngeal carcinoma; *CI* Confidence interval; *ADC* Apparent diffusion coefficient

### Classification model and performance

**Fig. 4:**
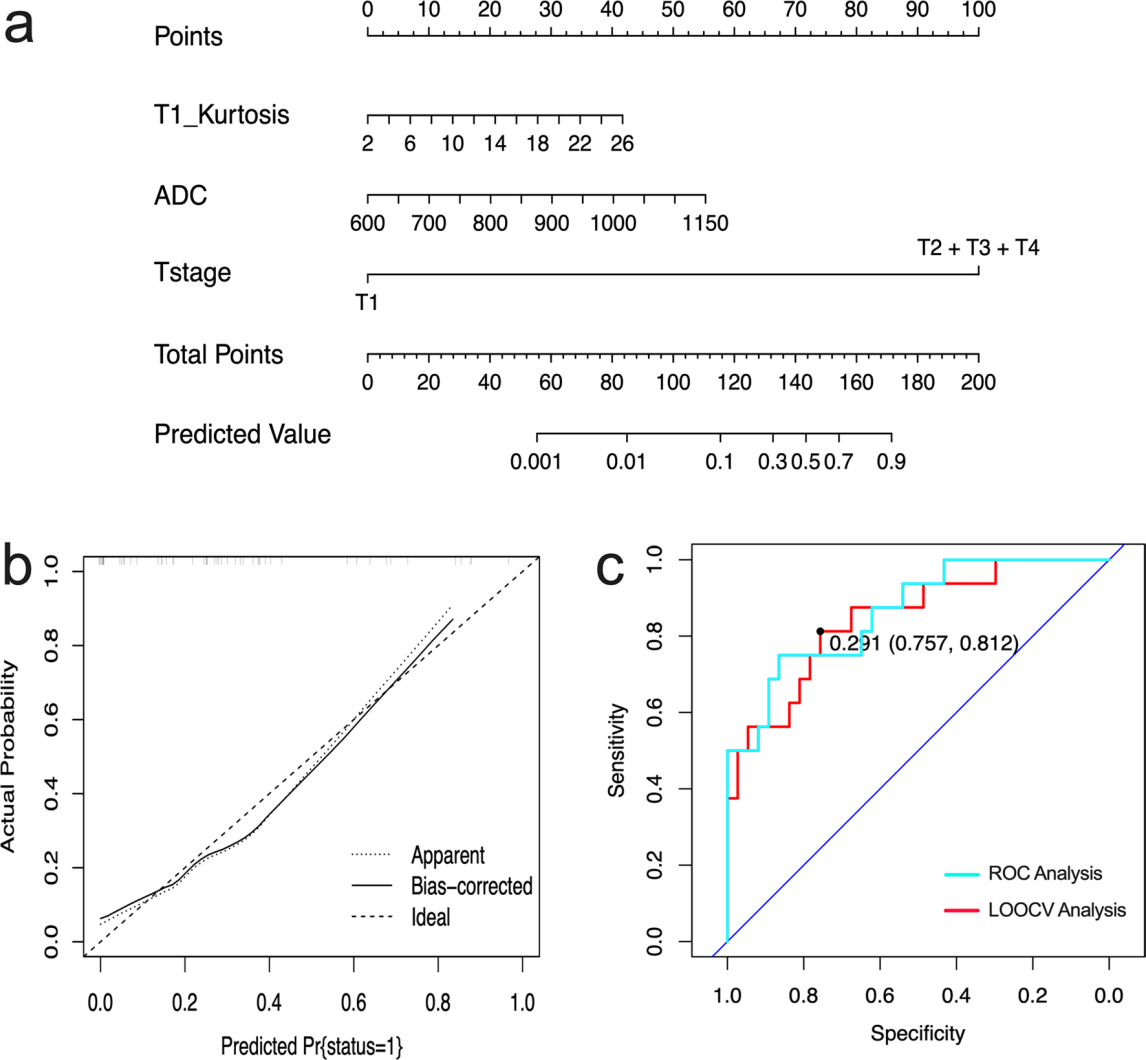
The performance of SyMRI + DWI + Clinic model in predicting 3-year disease progression of NPC. **a** A nomogram incorporating T1_Kurtosis, ADC_Mean, and T stage for predicting 3-year disease progression; **b** Calibration plot shows the relationship between the actual values and the predicted probability based on the nomogram; **c** ROC analysis and LOOCV analysis of SyMRI + DWI + Clinic model. *SyMRI* Synthetic magnetic resonance imaging; *DWI* Diffusion-weighted imaging; *NPC* Nasopharyngeal carcinoma; *ROC* Receiver operating characteristic; *LOOCV* Leave-one-out cross-validation

The disease progression group showed higher T1_Kurtosis and T1_Skewness, lower T2_10th, PD_10th, and PD_Mean than non-disease progression group (all *p* ≤ 0.041, Additional file 1: Table S1). T1_Kurtosis showed the best diagnostic performance among all SyMRI-derived parameters for distinguishing those two groups, with an AUC of 0.748 (Table 4). The combination of SyMRI, DWI, and Clinic can significantly improve the AUC when compared with DWI and Clinic (AUC: 0.635) alone with all *p* < 0.008 (Fig. 3). Interestingly, there was no significant difference between the SyMRI model and SyMRI + DWI + Clinic model (*p* = 0.092, Fig. 3). The nomogram and calibration curve are shown in Fig. 4. LOOCV analysis revealed that the bias-corrected AUC of SyMRI + DWI + Clinic model was 0.863 (95% CI 0.757, 0.969), as shown in Fig. 4.

**Table 4:** Performance of quantitative SyMRI and DWI parameters in the prediction of 3-year disease progression

	Cutoff value	AUC (95% CI)	Sensitivity (%)	Specificity (%)	Accuracy (%)	PPV	NPV
*SyMRI model*							
T1_Kurtosis	6.98	0.748 (0.590, 0.907)	81.3	64.9	73.6	0.625	0.756
*DWI model*							
ADC	868.00	0.716 (0.564, 0.868)	68.8	73.0	71.7	0.571	0.739
Clinic model T stage	T1 stage	0.635 (0.485, 0.785)	100.0	27.0	69.8	0	0.698
SyMRI + DWI + Clinic model T1_Kurtosis + ADC + T stage	0.29	0.846 (0.731, 0.961)	81.2	75.7	81.1	0.818	0.833

*SyMRI* Synthetic magnetic resonance imaging; *DWI* Diffusion-weighted imaging; *PD* Proton density; *ADC* Apparent diffusion coefficient; *AUC* Area under the curve; *95% CI* 95% confidence interval

Logit (SyMRI) = 0.18 × T1_Kurtosis − 2.471; Logit (SyMRI + DWI + Clinic) = 0.136 × T1_Kurtosis + 0.008 × ADC + 20.643 × T stage − 29.05

**Fig. 5:**
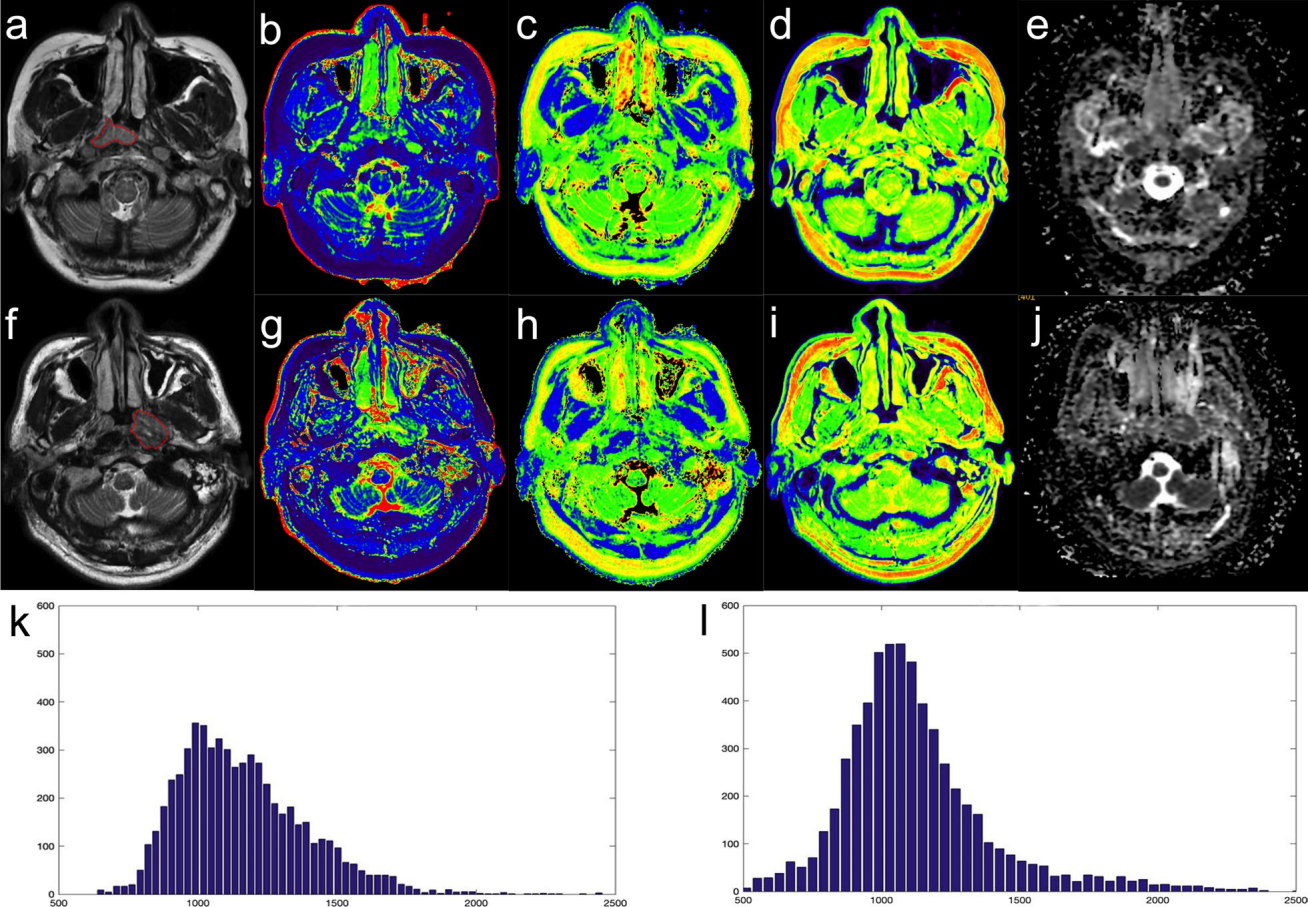
Representative pretreatment images of NPC patients without disease progression (**a**–**e**, **k**) and with disease progression (**f**–**j**, **i**). SyT2WI (**a**, **f**), T1 map (**b**, **g**), T2 map (**c**, **h**), PD map (**d**, **i**), ADC map (**e**, **j**), and histogram of T1 map (**k**, **l**) of NPC patients are shown. A 48-year-old woman with NPC (T1_Kurtosis: 4.34) showed no disease progression at 39.6 months after treatment initiation (**a**–**e**, **k**). A 56 year-old man with NPC (T1_Kurtosis: 7.03) showed disease progression at 25.2 months (**f**–**j**, **i**)

Representative pretreatment axial SyMRI images, T1, T2, PD, and ADC maps, and T1 histogram of NPC patients with and without disease progression are shown in Fig. 5.

## Discussion

In this study, we evaluated the prognostic value of histogram parameters extracted from pretreatment SyMRI in the assessment of patients at risk for 3-year disease progression. After adjusting for conventional clinical factors as confounding variables, the T1_Kurtosis and ADC remained significant predictors of PFS. The SyMRI + DWI + Clinic model significantly increased the diagnostic performance compared with DWI or Clinic single model. Particularly, there was no significant difference between SyMRI model and SyMRI + DWI + Clinic model, suggesting good prospects for SyMRI in the prognostic evaluation of tumors. As far as we know, this is the first study based on SyMRI to focus on long-term prognosis. Quantitative T1, T2, and PD values of SyMRI, which were not affected by different scanners and scan parameters, supply clinicians with an objective and reliable assessment method.

The benefit of SyMRI is twofold, providing relaxometry maps in a clinically feasible time and the ability to generate different contrast images in a single acquisition [17]. Quantitative T1, T2, and PD Mean values were reported to be lower in malignant tumors vs. benign tumors [20, 27, 28], and in poor prognostic relevant factors tumors vs. good prognostic relevant factors [20–23, 29]. Moreover, several histogram parameters, such as 10th Percentile, Kurtosis, Energy, Standard Deviation, and so on, were reported to predict tumor molecular subtypes [30] and lymph node metastasis (LNM) [31]. The research works [18, 19] in breast cancer found changes in T1 during treatment and T2_Standard Deviation were useful to predict pathological response after neoadjuvant therapy. However, treatment response in NPC is difficult to assess accurately due to inaccessibility to biopsy and minor residual and deeply seated abnormalities may be ignored [14]. Over 90% metastasis of NPC occurred in the first 3 years after treatment [32]. Therefore, our study set 3 years as a minimum follow-up time for surviving patients.

Currently, the intratumor heterogeneity has been reported to be associated with prognosis [33]. Histogram and texture analysis, as well as radiomics, were introduced for a comprehensive assessment of tumor characteristics. Previous studies found radiomics features based on conventional MRI could help predict disease progression in NPC [24, 32]. However, a recent study showed that among 177 radiomics features including shape, first-order and texture features, many radiomics features were redundant [34]. Besides, variations in acquisition and image reconstruction parameters can obscure underlying biological effects [35], and the radiomics feature selection is relatively instability [36]. Therefore, we investigated the value of SyMRI combined histogram analysis in the prognosis assessment of NPC. The percentile parameter is less influenced by random fluctuations than Mean value statistically [37], and Skewness of APT helps in discriminating 2-year disease-free survival in NPC [16], so four histogram parameters, including 10th Percentile, Mean, Kurtosis, and Skewness, were included in our study. 10th Percentile and Mean reflect signal intensity within the VOIs. Kurtosis and Skewness reflect histogram peakedness and asymmetry, which could indirectly describe the image gray-level heterogeneity [38]. The Kurtosis and Skewness are 3 and 0 when the data is Gaussian distribution. Higher Kurtosis indicates that the mass of the distribution is concentrated toward the tails rather than toward the mean, and positive Skewness indicates that there is a greater frequency of low quantitative values (the curve is skewed right).

Quantitative T1 value depends on the composition of tissue, such as macromolecule concentration, tissue water content, and proliferation level [21]. High levels of extracellular macromolecules and tissue water content result in higher T1 value. Besides, high T2 and PD values are reported in correlation with high extracellular water content [30, 39]. Our research demonstrated that T1_Kurtosis, T1_Skewness, T2_10th, and PD_Mean were correlated with PFS in NPC, and T1_Kurtosis was an independent prognostic factor after adjusting for confounding factors. Patients with high Kurtosis, which suggests the tumor is heterogeneous [40], exhibited worse PFS than those with low Kurtosis in our study. This finding supports the view that high tumor heterogeneity is usually associated with poor prognosis [41]. Similar results were found in previous studies [24, 38]. Lower contrast-enhanced T1WI-based Mean Absolute Deviation [24] and higher contrast-enhanced T1WI-based Uniformity [38] were the predictive factors for favorable PFS in NPC. The SyMRI studies in breast/rectal cancer also found lower Kurtosis of PD and T2 maps was associated with good prognostic relevant factors (such as low grade and without LNM) [30, 31]. Poor outcome is associated with primary tumors that have high stromal content, hypoxia, low proliferation, and decreased blood volume/flow [14, 42]. A decrease in tumor blood volume/flow as well as hypoxia induces more deoxyhemoglobin (paramagnetic properties) accumulation, and lower proliferation reduce T1 value [43]. A greater frequency of low T1 value in the disease progression group caused higher T1_Skewness (the histogram curve skew right), compared to the non-disease progression group. The Skewness parameter could comprehensively reflect intratumor features, not only with the change of value but also with heterogeneity characteristics. Similar results were found in previous studies based on DWI [44, 45]. Furthermore, the research in NPC [42, 46] found the response and good prognostic groups have abundant blood supply/volume, high microcirculation perfusion, and oxygen content than the non-response and worse groups. Increased tumor blood volume/flow and perfusion and low stromal content in the non-disease progression group increase the extracellular water space and thus raise T1, T2, and PD values [43, 47]. This may be the major reason for high T2_10th, PD_10th, and PD_Mean in the non-disease progression group.

ADC value, which is derived from DWI, noninvasively reflects the Brownian motion of water molecules. In our study, we found ADC_Mean was an independent prognostic factor in PFS of NPC and low ADC_Mean was correlated with non-disease progression survival, which was similar to previous studies [48, 49]. Tumor lesions with low ADC have lower stromal content and higher cell density, suggesting that relatively abundant blood supply therefore result in higher radiosensitivity [49, 50] and favorable survival. Moreover, a high ADC value level also indicates invasive biological features of the tumor, leading to the high possibility of local disease progression [48].

One of the advantages of SyMRI is that it can directly reflect tissue intrinsic characteristics provided by quantitative T1, T2, and PD maps without contrast agents. Freed from different scan parameters and machines, SyMRI provides a robust, objective, and reliable method in the clinic. There are some limitations in this study. First, the sample is relatively small, but all patients in this study were included consecutively and stable results were confirmed by two statistical methods. Multiple centers with large samples are needed in the future. Second, pretreatment EBV DNA level was a useful predictor of disease progression [49]. But EBV level was not significant in our study partly because of the EBV cutoff value we chose and large interlaboratory variability existed in EBV analysis. New techniques such as digital PCR or next-generation sequencing and the simultaneous analysis of two EBV sequences can be of great benefit for assay harmonization [1]. Third, no patient in T1 stage occurred with disease progression and the follow-up time was relatively short in PFS prediction. However, T1-stage patients with NPC do have good PFS and survival quality. And our study focused on whether SyMRI could predict 3-year disease progression. A clear endpoint for the development of disease progression (with or without) has advantages over an unclear endpoint such as survival [45]. Ai et al. [45] found seven NPC patients in stage T1-2 were diagnosed with distant metastasis during 5-year follow-up. Therefore, longer follow-up is needed in further research.

## Conclusion

Several quantitative parameters of SyMRI (T1_Kurtosis, T1_Skewness, T2_10th, and PD_Mean) were demonstrated efficient in predicting PFS in NPC, and T1_Kurtosis showed high diagnostic efficiency in predicting 3-year disease progression. Furthermore, the SyMRI + DWI + Clinic model showed outstanding prediction efficiency in disease progression before initial treatment. These findings demonstrated the feasibility of SyMRI in prognostic assessment and required further research before it can be translated into clinical practice.

## Supplementary Information

**Additional file 1.** Supplementary Tables and Fig.

## Data Availability

The datasets used and analyzed during the current study are available from the corresponding author upon reasonable request.

## Abbreviations

95% CI: 95% confidence interval
ADC: Apparent diffusion coefficient
AUC: Area under the curve
DWI: Diffusion-weighted imaging
EBV: Epstein–Barr virus
ICC: Interclass correlation
LOOCV: Leave-one-out cross-validation
NPC: Nasopharyngeal carcinoma
PD: Proton density
PFS: Progression-free survival
ROC: Receiver operating characteristic
SyMRI: Synthetic magnetic resonance imaging
T2WI: T2-weighted imaging
VOI: Volume of interest

## References

[1] Chen YP, Chan ATC, Le QT, Blanchard P, Sun Y, Ma J (2019). Nasopharyngeal carcinoma. Lancet.

[2] Lee AW, Ma BB, Ng WT, Chan AT (2015). Management of nasopharyngeal carcinoma: current practice and future perspective. J Clin Oncol.

[3] You R, Zou X, Wang SL (2015). New surgical staging system for patients with recurrent nasopharyngeal carcinoma based on the AJCC/UICC rTNM classification system. Eur J Cancer.

[4] Zhang L, Huang Y, Hong S (2016). Gemcitabine plus cisplatin versus fluorouracil plus cisplatin in recurrent or metastatic nasopharyngeal carcinoma: a multicentre, randomised, open-label, phase 3 trial. Lancet.

[5] Zhang L, Dong D, Li H (2019). Development and validation of a magnetic resonance imaging-based model for the prediction of distant metastasis before initial treatment of nasopharyngeal carcinoma: a retrospective cohort study. EBioMedicine.

[6] Xu C, Chen YP, Liu X (2017). Establishing and applying nomograms based on the 8th edition of the UICC/AJCC staging system to select patients with nasopharyngeal carcinoma who benefit from induction chemotherapy plus concurrent chemoradiotherapy. Oral Oncol.

[7] Lee VH, Kwong DL, Leung TW (2019). The addition of pretreatment plasma Epstein–Barr virus DNA into the eighth edition of nasopharyngeal cancer TNM stage classification. Int J Cancer.

[8] Guo R, Tang LL, Mao YP (2019). Proposed modifications and incorporation of plasma Epstein–Barr virus DNA improve the TNM staging system for Epstein–Barr virus-related nasopharyngeal carcinoma. Cancer.

[9] Zhou GQ, Tang LL, Mao YP (2012). Baseline serum lactate dehydrogenase levels for patients treated with intensity-modulated radiotherapy for nasopharyngeal carcinoma: a predictor of poor prognosis and subsequent liver metastasis. Int J Radiat Oncol Biol Phys.

[10] Xia WX, Zhang HB, Shi JL (2013). A prognostic model predicts the risk of distant metastasis and death for patients with nasopharyngeal carcinoma based on pre-treatment serum C-reactive protein and N-classification. Eur J Cancer.

[11] Xiong Y, Shi LL, Zhu LS, Ding Q, Ba L, Peng G (2021). Prognostic efficacy of the combination of the pretreatment systemic immune-inflammation index and Epstein–Barr virus DNA status in locally advanced nasopharyngeal carcinoma patients. J Cancer.

[12] Le QT, Zhang Q, Cao H (2013). An international collaboration to harmonize the quantitative plasma Epstein–Barr virus DNA assay for future biomarker-guided trials in nasopharyngeal carcinoma. Clin Cancer Res.

[13] Wang G, He L, Yuan C, Huang Y, Liu Z, Liang C (2018). Pretreatment MR imaging radiomics signatures for response prediction to induction chemotherapy in patients with nasopharyngeal carcinoma. Eur J Radiol.

[14] Qamar S, King AD, Ai QH (2020). Pre-treatment intravoxel incoherent motion diffusion-weighted imaging predicts treatment outcome in nasopharyngeal carcinoma. Eur J Radiol.

[15] Zhao DW, Fan WJ, Meng LL (2021). Comparison of the pre-treatment functional MRI metrics' efficacy in predicting Locoregionally advanced nasopharyngeal carcinoma response to induction chemotherapy. Cancer Imaging.

[16] Qamar S, King AD, Ai QH (2020). Pre-treatment amide proton transfer imaging predicts treatment outcome in nasopharyngeal carcinoma. Eur Radiol.

[17] Konar AS, Paudyal R, Shah AD (2022). Qualitative and quantitative performance of magnetic resonance image compilation (MAGiC) method: an exploratory analysis for head and neck imaging. Cancers (Basel).

[18] Du S, Gao S, Zhao R (2022). Contrast-free MRI quantitative parameters for early prediction of pathological response to neoadjuvant chemotherapy in breast cancer. Eur Radiol.

[19] Matsuda M, Fukuyama N, Matsuda T (2022). Utility of synthetic MRI in predicting pathological complete response of various breast cancer subtypes prior to neoadjuvant chemotherapy. Clin Radiol.

[20] Cui Y, Han S, Liu M (2020). Diagnosis and grading of prostate cancer by relaxation maps from synthetic MRI. J Magn Reson Imaging.

[21] Cai Q, Wen Z, Huang Y (2021). Investigation of synthetic magnetic resonance imaging applied in the evaluation of the tumor grade of bladder cancer. J Magn Reson Imaging.

[22] Zhao L, Liang M, Wu PY, Yang Y, Zhang H, Zhao X (2021). A preliminary study of synthetic magnetic resonance imaging in rectal cancer: imaging quality and preoperative assessment. Insights Imaging.

[23] Ma L, Lian S, Liu H (2022). Diagnostic performance of synthetic magnetic resonance imaging in the prognostic evaluation of rectal cancer. Quant Imaging Med Surg.

[24] Du R, Lee VH, Yuan H (2019). Radiomics model to predict early progression of nonmetastatic nasopharyngeal carcinoma after intensity modulation radiation therapy: a multicenter study. Radiol Artif Intell.

[25] Park JE, Kim HS, Park KJ, Choi CG, Kim SJ (2015). Histogram analysis of amide proton transfer imaging to identify contrast-enhancing low-grade brain tumor that mimics high-grade tumor: increased accuracy of MR perfusion. Radiology.

[26] Park JE, Kim HS, Park KJ, Kim SJ, Kim JH, Smith SA (2016). Pre- and posttreatment glioma: comparison of amide proton transfer imaging with MR spectroscopy for biomarkers of tumor proliferation. Radiology.

[27] Meng T, He H, Liu H (2021). Investigation of the feasibility of synthetic MRI in the differential diagnosis of non-keratinising nasopharyngeal carcinoma and benign hyperplasia using different contoured methods for delineation of the region of interest. Clin Radiol.

[28] Meng T, He N, He H (2020). The diagnostic performance of quantitative mapping in breast cancer patients: a preliminary study using synthetic MRI. Cancer Imaging.

[29] Li S, Liu J, Zhang F (2020). Novel T2 mapping for evaluating cervical cancer features by providing quantitative T2 maps and synthetic morphologic images: a preliminary study. J Magn Reson Imaging.

[30] Li Q, Xiao Q, Yang M (2021). Histogram analysis of quantitative parameters from synthetic MRI: correlations with prognostic factors and molecular subtypes in invasive ductal breast cancer. Eur J Radiol.

[31] Zhao L, Liang M, Shi Z, Xie L, Zhang H, Zhao X (2021). Preoperative volumetric synthetic magnetic resonance imaging of the primary tumor for a more accurate prediction of lymph node metastasis in rectal cancer. Quant Imaging Med Surg.

[32] Bao D, Liu Z, Geng Y (2022). Baseline MRI-based radiomics model assisted predicting disease progression in nasopharyngeal carcinoma patients with complete response after treatment. Cancer Imaging.

[33] Mroz EA, Tward AD, Pickering CR, Myers JN, Ferris RL, Rocco JW (2013). High intratumor genetic heterogeneity is related to worse outcome in patients with head and neck squamous cell carcinoma. Cancer.

[34] Berenguer R, Pastor-Juan MDR, Canales-Vázquez J (2018). Radiomics of CT features may be nonreproducible and redundant: influence of CT acquisition parameters. Radiology.

[35] Gillies RJ, Kinahan PE, Hricak H (2016). Radiomics: images are more than pictures, they are data. Radiology.

[36] Wong LM, Ai QYH, Zhang R, Mo F, King AD (2022). Radiomics for discrimination between early-stage nasopharyngeal carcinoma and benign hyperplasia with stable feature selection on MRI. Cancers (Basel).

[37] Chung WJ, Kim HS, Kim N, Choi CG, Kim SJ (2013). Recurrent glioblastoma: optimum area under the curve method derived from dynamic contrast-enhanced T1-weighted perfusion MR imaging. Radiology.

[38] Mao J, Fang J, Duan X (2019). Predictive value of pretreatment MRI texture analysis in patients with primary nasopharyngeal carcinoma. Eur Radiol.

[39] Mezer A, Rokem A, Berman S, Hastie T, Wandell BA (2016). Evaluating quantitative proton-density-mapping methods. Hum Brain Mapp.

[40] Noda Y, Tomita H, Ishihara T (2022). Prediction of overall survival in patients with pancreatic ductal adenocarcinoma: histogram analysis of ADC value and correlation with pathological intratumoral necrosis. BMC Med Imaging.

[41] de Bruin EC, McGranahan N, Mitter R (2014). Spatial and temporal diversity in genomic instability processes defines lung cancer evolution. Science.

[42] Chan SC, Yeh CH, Chang JT, Chang KP, Wang JH, Ng SH (2021). Combing MRI perfusion and (18)F-FDG PET/CT metabolic biomarkers helps predict survival in advanced nasopharyngeal carcinoma: a prospective multimodal imaging study. Cancers (Basel).

[43] McSheehy PM, Weidensteiner C, Cannet C (2010). Quantified tumor t1 is a generic early-response imaging biomarker for chemotherapy reflecting cell viability. Clin Cancer Res.

[44] Law BK, King AD, Bhatia KS (2016). Diffusion-weighted imaging of nasopharyngeal carcinoma: Can pretreatment DWI predict local failure based on long-term outcome?. AJNR Am J Neuroradiol.

[45] Ai QY, King AD, Law BK (2017). Diffusion-weighted imaging of nasopharyngeal carcinoma to predict distant metastases. Eur Arch Otorhinolaryngol.

[46] Sun Z, Hu S, Xue Q, Jin L, Huang J, Dou W (2021). Can 3D pseudo-continuous arterial spin labeling perfusion imaging be applied to predict early response to chemoradiotherapy in patients with advanced nasopharyngeal carcinoma?. Radiother Oncol.

[47] Le Bihan D, Breton E, Lallemand D, Aubin ML, Vignaud J, Laval-Jeantet M (1988). Separation of diffusion and perfusion in intravoxel incoherent motion MR imaging. Radiology.

[48] Huang TX, Lu N, Lian SS (2019). The primary lesion apparent diffusion coefficient is a prognostic factor for locoregionally advanced nasopharyngeal carcinoma: a retrospective study. BMC Cancer.

[49] Liu LT, Guo SS, Li H (2021). Percent change in apparent diffusion coefficient and plasma EBV DNA after induction chemotherapy identifies distinct prognostic response phenotypes in advanced nasopharyngeal carcinoma. BMC Cancer.

[50] Vidiri A, Marzi S, Gangemi E (2019). Intravoxel incoherent motion diffusion-weighted imaging for oropharyngeal squamous cell carcinoma: correlation with human papillomavirus status. Eur J Radiol.

