# Supplementary material for: Pretreatment synthetic magnetic resonance imaging predicts disease progression in nonmetastatic nasopharyngeal carcinoma after intensity modulation radiation therapy
Source: Insights Imaging. 2023 Apr 5;14:59. doi: 10.1186/s13244-023-01411-y (PMC10073373; doi:10.1186/s13244-023-01411-y)
Supplement: Supplementary file 1 — Additional file 1. Supplementary Tables and Fig. [file 13244_2023_1411_MOESM1_ESM.pdf]

ELECTRONIC SUPPLEMENTARY MATERIAL

Pre-treatment Synthetic Magnetic Resonance Imaging predicts disease progression in nonmetastatic nasopharyngeal carcinoma after intensity modulation radiation therapy

Supplementary Table 1 Comparisons of quantitative SyMRI and DWI parameters between NPC patients with and without disease progression

Parameters	Disease progression group (n = 16)	Non-disease progression group (n = 37)	P value	ICC	AUC (95% CI)	Sensitivity (%)	Specificity (%)	Accuracy (%)	PPV	NPV
SyMRI										
T1_10th	1192.22 ± 180.13	1196.48 ± 98.03	0.462	0.912	NA	NA	NA	NA	NA	NA
T1_Mean	1471.34 ± 244.23	1511.04 ± 156.13	0.237	0.901	NA	NA	NA	NA	NA	NA
**T1_Kurtosis**	**12.14 ± 7.42**	**7.13 ± 3.42**	**0.004**	0.982	**0.748 (0.590, 0.907)**	**81.3**	**64.9**	**73.6**	**0.625**	**0.756**
**T1_Skewness**	**1.84 ± 0.91**	**1.35 ± 0.53**	**0.018***	0.985	**0.704 (0.539, 0.870)**	**68.9**	**70.3**	**73.6**	**0.667**	**0.745**
**T2_10th**	**68.64 ± 8.65**	**72.69 ± 5.08**	**0.038**	0.978	**0.679 (0.506, 0.852)**	**56.3**	**83.8**	**77.4**	**0.833**	**0.766**
T2_Mean	83.45 ± 9.72	86.94 ± 6.43	0.128*	0.983	NA	NA	NA	NA	NA	NA
T2_Kurtosis	17.51 ± 21.26	10.76 ± 9.64	0.397	0.989	NA	NA	NA	NA	NA	NA
T2_Skewness	1.92 ± 1.91	1.39 ± 1.00	0.482	0.990	NA	NA	NA	NA	NA	NA
**PD_10th**	**77.95 ± 3.60**	**80.24 ± 3.50**	**0.041***	0.948	**0.671 (0.518, 0.825)**	**51.4**	**81.2**	**69.8**	**0.500**	**0.714**
**PD_Mean**	**88.00 ± 3.24**	**90.30 ± 2.90**	**0.014**	0.891	**0.698 (0.552, 0.843)**	**93.8**	**37.8**	**71.7**	**0.571**	**0.739**
PD_Kurtosis	4.01 ±1.43	3.68 ± 1.35	0.287	0.980	NA	NA	NA	NA	NA	NA
PD_Skewness	-0.57 ± 0.43	-0.58 ± 0.46	0.925*	0.983	NA	NA	NA	NA	NA	NA
DWI										
**ADC**	**897.09 ± 124.20**	**804.30 ± 115.97**	**0.017***	NA	**0.716 (0.564, 0.868)**	**68.8**	**73.0**	**71.7**	**0.571**	**0.739**

* indicates independent sample t test.

SyMRI, synthetic magnetic resonance imaging; DWI, diffusion-weighted imaging; PD, proton density; ADC, apparent diffusion coefficient; AUC, area under curve; 95% CI, 95% confidence interval; NA, not applicable.

Supplementary Table 2 Details of 16 Patients with recurrence

Patients	Local Recurrence		Regional Recurrence		Distant Metastasis	
	Imaging	Biopsy	Imaging	Biopsy	Site	Imaging
1	MRI + CT + PET-CT	(+)			Lung	CT + PET-CT
2	MRI + CT + PET-CT	(+)			Brain	MRI + PET-CT
3	MRI+ CT	(+)				
4	MRI+ CT	(+)				
5	MRI+ CT	(+)				
6	MRI+ CT	(+)				
7	No abnormal findings	(+)				
8			MRI + CT + PET-CT	(+)		
9			MRI + CT	(+)		
10			MRI + CT	(+)		
11					Bone	MRI +PET-CT
12					Bone	MRI + Bone scan + CT
13					Liver	MRI + CT + US
14					Liver	MRI + CT + US
15					Brain	MRI
16					Lung	CT

Supplementary Fig.1 The distribution of the C-index of SyMRI + DWI + Clinic model (a) and SyMRI model (b) using bootstrap (n = 1000).

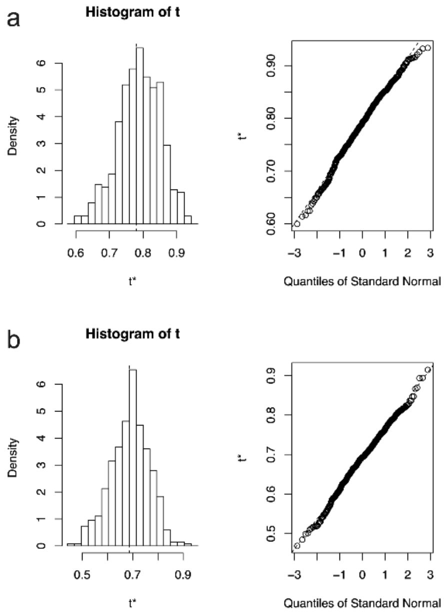

Insights Imaging (2023) Yang F, Wei H, Li X et al.
